# Retreatment strategies following Small Incision Lenticule Extraction (SMILE): In vivo tissue responses

**DOI:** 10.1371/journal.pone.0180941

**Published:** 2017-07-14

**Authors:** Andri K. Riau, Yu-Chi Liu, Chris H. L. Lim, Nyein C. Lwin, Ericia P. Teo, Gary H. Yam, Donald T. Tan, Jodhbir S. Mehta

**Affiliations:** 1 Tissue Engineering and Stem Cell Group, Singapore Eye Research Institute, Singapore; 2 Singapore National Eye Centre, Singapore; 3 Royal Melbourne Hospital, Melbourne, Australia; 4 Department of Ophthalmology, National University Health System, Singapore; 5 Ophthalmology Academic Clinical Program, Duke-NUS Graduate Medical School, Singapore; 6 Department of Clinical Sciences, Duke-NUS Graduate Medical School, Singapore; Bascom Palmer Eye Institute, UNITED STATES

## Abstract

With any refractive correction, including Small Incision Lenticule Extraction (SMILE), there may be a residual refractive error that requires a retreatment. Here, we investigated the tissue responses following various retreatment procedures in a rabbit model of SMILE. All rabbits underwent a -6.00D correction with SMILE. Two weeks later, they underwent -1.00D enhancement by: (i) VisuMax Circle, followed by excimer ablation (S+C); (ii) secondary SMILE anterior to the primary procedure (S+SE); or (iii) surface ablation (S+P), and were examined for 28 days. S+P induced corneal edema and haze, and more CD11b- (23±6 cells) and TUNEL-positive (36±4 cells) cells in the central stromal superficial layers early post-operatively (*p*<0.001 compared to other procedures). The corneas appeared normal on day 28 after S+P, but had a lower number of keratocytes near the laser ablated plane compared to other procedures. S+SE and S+C did not induce corneal haze and resulted similar level of fibronectin. However, S+C resulted in more inflammatory (10±2 cells; *p* = 0.001) and apoptotic cells (25±2 cells; *p*<0.001) compared to S+SE (7±1 inflammatory cells and 21±3 apoptotic cells) early post-operatively. In conclusion, each SMILE retreatment method resulted in unique tissue responses. S+SE offers advantages, such as minimal inflammation and cell death, as well as maintaining a ‘flap-less’ surgery, over other procedures. However, depending on the degree of enhancement, the lenticule may become too thin to be extracted and the procedure becomes more difficult to perform than S+C and S+P. S+P can maintain corneal integrity by avoiding flap creation and is technically more simple to perform than the others, but the surgery needs to be supplemented with mitomycin-C in order to reduce inflammation and modulate better wound healing.

## Introduction

The earlier variant of refractive lenticule extraction (ReLEx), femtosecond lenticule extraction (FLEx), is a procedure in which a hinged corneal flap was created by FSL, the flap was then lifted, and the refractive lenticule stripped away [1,2]. The current variant, small incision lenticule extraction (SMILE), represents a flap-less procedure utilizing a small incision, whereby the refractive lenticule is extracted through a keyhole incision [3,4]. Both variants of ReLEx have been demonstrated to be safe, with clinical results comparable to LASIK [2–5]. Moreover, as there is no flap created in the procedure, SMILE has been shown to induce less corneal nerve damage and result in faster recovery of post-operative corneal sensitivity and hence fewer incidence of dry eye than LASIK [6,7]. In addition, SMILE also results in a biomechanically more stable cornea [8,9].

Enhancement rates after LASIK range from 5% to 28% [10–14]. In LASIK, the original flap can simply be re-lifted for a retreatment or alternatively, a surface ablation or recutting of a new flap can be performed [14,15]. However, in a flap-less procedure, such as SMILE, a different approach needs to be employed. Retreatment rates after SMILE have not been extensively reported, but Reinstein et al. reported a 4% enhancement rate after low myopic treatment [16]. It cannot be completely ruled out that some SMILE patients may have a residual refractive error from initial under/over correction, induced astigmatism, or regression [17,18]. If the residual refractive error is significant enough, these patients may seek further refractive correction or enhancement. While flap re-lifting and refractive enhancement can be easily achieved in LASIK, the lack of a flap in SMILE poses a unique challenge when enhancement is indicated.

We have previously demonstrated the application of VisuMax Circle pattern (Carl Zeiss Meditec, Jena, Germany) to create a corneal flap for refractive enhancement after SMILE in a rabbit model [19]. In a recent study, Chansue et al. reported successful clinical cases in 28 eyes that underwent SMILE enhancement utilizing the same Circle pattern [20]. In an alternative approach, Ivarsen and Hjortdal reported the appearance of haze in 5 eyes that underwent surface ablation enhancement [21]. Due to the variability in reported clinical outcomes following several SMILE enhancement techniques, we set out to investigate the corneal wound healing and inflammatory responses by immunohistochemistry, in vivo confocal microscopy, and anterior segment-optical coherence tomography (AS-OCT) following SMILE retreatment in a rabbit experimental model.

## Materials and methods

### Animals

Fifteen 12- to 15-week-old New Zealand White rabbits (3–4 kg body weight) were purchased from InVivos, Singapore and used in this study. The rabbits were housed in adjoining individual stainless steel cages allowing social interactions. The room environment was continuously controlled for temperature (24±2^°^C), humidity (50±20%), light cycle (12 hours light and 12 hours dark), and air change (10–15 air changes/hour). Before any surgery or pre- and post-operative examination, the rabbits were anesthetized with ketamine hydrochloride (50 mg/kg intramuscularly; Parnell Laboratories, Alexandria, Australia) and xylazine hydrochloride (5 mg/kg intramuscularly; Troy Laboratories, Smithfield, Australia). Topical xylocaine (AstraZeneca, London, UK) was applied on the ocular surface before surgeries. Following surgeries, a one-time subconjunctival injection of gentamicin sulphate (40 mg/ml; Shin Poong Pharmaceutical, Seoul, South Korea) and dexamethasone sodium phosphate (4 mg/ml; Hospira, Lake Forest, IL) of 0.1 ml each was administered. Euthanasia was performed following administration of anesthesia via an intracardiac bolus injection of sodium pentobarbitone (Jurox, Rutherford, Australia).

The protocol was approved by the Institutional Animal Care and Use Committee of SingHealth, Singapore. The animals were treated according to the guidelines of the Association for Research in Vision and Ophthalmology’s Statement for the Use of Animals in Ophthalmic and Vision Research. The experimental design was summarized in S1 Fig. The rabbits underwent primary SMILE for -6.00D spherical correction bilaterally. Pertaining guidelines for bilateral ocular survival surgery could be found in the ARVO's Statement for the Use of Animals in Ophthalmic and Visual Research (available at: http://www.arvo.org/about_arvo/policies/statement_for_the_use_of_animals_in_ophthalmic_and_visual_research/#factors). At no point in time during the study, the rabbits suffered from bilateral visual deprivation. Continuous care (24 hours/7 days) was provided throughout the study to ensure prompt intervention when needed. The rabbits were monitored carefully for signs of eating behavioural and activity changes daily. Previous studies had shown that SMILE procedure did not induce corneal haze in rabbits [1,7,19]. Hence, this study was designed as such that all rabbits that underwent bilateral corneal surgeries had SMILE as the enhancement method on the left eye. Two weeks after the primary SMILE, the rabbits were subjected to one of the following enhancement procedures: (i) -1.00D surface ablation, and these rabbits were euthanized 1 day or 28 days post-operatively (the groups were referred to as S+P day 1 and S+P day 28, respectively); (ii) secondary -1.00D SMILE performed anterior to the primary procedure, and these rabbits were euthanized on post-operative day 1 (S+SE day 1 group) or post-operative day 28 (S+SE day 28 group); (iii) secondary SMILE, but the refractive lenticule was not extracted (S+SN day 1 group); and (iv) flap creation by VisuMax Circle software, followed by -1.00D stromal excimer ablation, and these rabbits were euthanized 1 day post-operatively (S+C day 1 group). The S+SN group was used to study the effect of FSL delivery on the stromal layers, without being subjected to any intrastromal manipulation lenticule extraction. In addition to the treatment groups stated above, -1.00D photorefractive keratectomy (PRK) was performed on 6 rabbit corneas to compare the tissue responses after S+P with that after surface ablation alone. These rabbits were euthanized on post-operative days 1 and 28 (referred to as PRK day 1 and PRK day 28 groups, respectively). All groups consisted of 3 rabbit eyes. Untreated eyes were used as negative controls. Day 1 follow-up was performed to document early wound healing process, whereas day 28 follow-up was chosen as a time point to document late tissue response (corneal haze) to PRK procedure that was normally seen in rabbits [22,23].

### Small incision lenticule extraction procedure

SMILE was performed using the VisuMax FSL system (Carl Zeiss Meditec). All rabbits underwent a -6.00D spherical correction. Once suction was applied, the main refractive and non-refractive femtosecond incisions were performed in the following optimized sequence: the posterior surface of the lenticule (spiral in pattern), the anterior surface of the lenticule (spiral out pattern), followed by a 3-mm vertical incision to the corneal surface placed superiorly. The diameter and depth of the cap was set at 7.5 mm and 160 μm, respectively. The diameter of the lenticule (equating to the optical zone) was 6.5 mm. This resulted in a 0.5 mm-wide clearance zone on each side (zone between the circumference of corneal cap and optical zone). The FSL parameters were: 200 nJ power for lenticule, lenticule side cut, cap and cap side cut, and side cut angle of 90^o^. The spot distance and tracking spacing were set at 3 μm/ 3 μm for the lenticule, 2 μm/ 2 μm for the lenticule side cut, 3 μm/ 3 μm for the cap, and 2 μm/ 2 μm for the cap side cut.

Secondary SMILE, performed in S+SE and S+SN groups, was carried out as described above, with the spherical correction and depth of corneal cap adjusted to -1.00D and 110 μm, respectively. Following completion of the laser sequence, a Seibel spatula (Rhein Medical Inc., Petersburg, FL) was inserted into the vertical pocket incision to access the lamellar plane of dissection. A lamellar dissector (Asico, Westmont, IL) was used to release adhesions of the anterior portion of the lenticule from the overlying stroma and then the posterior surface of the lenticule from the underlying stroma. Once the lenticule was free from both surfaces, a co-axial Tan DSAEK forceps (Asico) was used to grasp the lenticule and extract it from the corneal stromal bed. Finally, a 24-gauge cannula was used to flush the pocket insertion with balanced salt solution.

### Flap creation by Circle pattern and excimer laser ablation

The VisuMax Circle pattern D, previously determined to be the optimal pattern used to convert the SMILE pocket into a corneal flap [19], was employed here. The FSL parameters were: 7.9 mm for outer diameter, 160 μm for side cut depth, 90^o^ side cut angle, superior hinge position, 7.0 mm for junction diameter, 150 μm for junction upper depth, and 170 μm for junction lower depth. Following completion of the laser sequence, a Sinskey hook (Rhein Medical Inc.) was used to create a small incision along the furrow of the flap side cut near the hinge. A Seibel spatula (Rhein Medical Inc.) was inserted under the flap edge through the incision to create an access from the lamellar ring to the original cap-stromal bed interface. A lamellar dissector (Asico) was then inserted through the intrastromal tunnel to gently release the flap-bed and remaining flap-lamellar ring adhesions. A Seibel spatula was re-inserted under the flap, flap adhesions were released completely by sweeping under the flap, and the flap was finally lifted.

After the flap was lifted, the underlying stroma received a 6.5-mm optical zone ablation using a Technolas excimer laser (Bausch & Lomb, Rochester, NY) with the following laser parameters: 2.0 μm spot size diameter, 120 mJ/cm^2^ pulse energy density, and -1.00D spherical correction. Once the flap was repositioned, a bandage contact lens (Bausch & Lomb) was applied and the eyelid was closed with a temporary tarsorraphy using a 6–0 silk suture.

### Photorefractive keratectomy (surface ablation) procedure

The corneal epithelium was first scraped with a #64 Beaver blade (Becton-Dickinson, Franklin Lakes, NJ), with an exception of a 0.5 mm rim at the limbus. After epithelial scraping, the underlying stroma received a 6.5-mm optical zone ablation using a Technolas excimer laser (Bausch & Lomb) with following laser parameters: 2.0 μm spot size diameter, 120 mJ/cm^2^ pulse energy density, and -1.00D spherical correction. In order to investigate natural tissue response to the laser procedure and maintain consistency in prophylactic treament in all experimental groups, no mitomycin C (MMC) was administered to the rabbits treated with PRK.

### Pre- and post-operative corneal imaging

Slit lamp photographs, AS-OCT and in vivo confocal microscopy scans were taken prior to primary SMILE, 1 day after primary SMILE, and 1 day after refractive enhancement. Slit lamp photographs were taken with a Zoom Slit Lamp NS-2D (Righton, Tokyo, Japan) and observed by a trained ophthalmologist (YCL). Signs of corneal haze, striae, and circumferential bands due to laser incision were carefully examined. AS-OCT was performed using the RTVue Fourier-Domain OCT (Optovue, Fremont, CA). The system was first adjusted to position the vertex at the center of the AS-OCT image and then slowly moved away until the vertical white beam was barely seen before the image was captured. The central corneal thickness (CCT) was taken at the center (0.0 mm) and at 1 mm either side of the center (+1.0 mm, -0.1 mm). The mean of the three measurements was reported. En-face images of the cornea were captured using an in vivo confocal microscope (Heidelberg retina tomography HRT3; Heidelberg Engineering GmbH, Heidelberg, Germany). A carbomer gel (Vidisic; Mann Pharma, Berlin, Germany) was used as immersion fluid. In vivo confocal micrographs were analyzed with the Heidelberg Eye Explorer version 1.5.1 software (Heidelberg Engineering GmbH). Semi-quantitative analysis of the reflectivity level of the secondary keratotomy site was performed by measuring the mean grey value of the reflective particles using the ImageJ software, as previously described [1]. Confocal scans at 20 μm posterior of the secondary keratotomy site were captured and visible keratocytes were counted.

### Immunohistochemistry and TUNEL assay

Cryosections (8 μm in thickness) of the rabbit corneas were fixed with 4% paraformaldehyde (Sigma, St. Louis, MI) for 15 minutes, washed with 0.01M PBS (1st Base, Singapore), blocked with 4% bovine serum albumin (Sigma) in 0.01M PBS, 0.15% Triton X-100 (Sigma) for 1 hour, and incubated with either mouse monoclonal antibody against cellular fibronectin (Millipore) diluted at 1:400, mouse monoclonal antibody against CD11b (BD Pharmingen) diluted at 1:100 in blocking solution, or with pre-diluted mouse monoclonal antibody against Ki-67 (Invitrogen, Carlsbad, CA) at 4^°^C overnight. After washing with 0.01M PBS, the sections were incubated with goat anti-mouse Alexa Fluor 488-conjugated secondary antibody (Invitrogen) at room temperature for 1 hour. Slides were then mounted with UltraCruz Mounting Medium containing DAPI (Santa Cruz Biotechnology, Santa Cruz, CA). For negative controls, non-immune serum was used in place of the specific primary antibody. Sections were observed and imaged with a Zeiss AxioImager Z1 fluorescence microscope (Carl Zeiss, Oberkochen, Germany).

To detect apoptotic cells, a fluorescence-based TUNEL (terminal deoxynucleotidyl transferase dUTP nick end labeling) assay (In Situ Cell Death Detection Kit, Roche Applied Science, Indianapolis, IN) was used according to the manufacturer’s instructions. The staining was then viewed with Zeiss AxioImager Z1 fluorescence microscope (Carl Zeiss). Quantification of CD11b- and TUNEL-positive cells were performed on 3 different samples (from 3 different fields of view of each sample) in each experimental group. Cell count was carried out on random cryosections of the central cornea, which had been marked during the tissue embedding process in OCT compound. Only TUNEL-positive cells that appeared in the corneal stroma were counted and considered to be affected by the enhancement procedure. Apoptotic cells, detected in the superficial layer of the corneal epithelium, were considered to be cells that were undergoing natural cell death process [24].

### Statistical analysis

Data were expressed as mean ± standard deviation (SD). Comparison of day 1 tissue responses between groups was analyzed by using one-way ANOVA and a post hoc Tukey test. Comparison between day 1 and 28 tissue responses within the same group was analyzed by independent samples t-test. A value of *p*<0.05 was considered to be statistically significant. All statistical analysis was performed using SPSS software (version 17.0, SPSS Inc., Chicago, IL).

## Results

### Slit lamp examination

**Fig 1:**
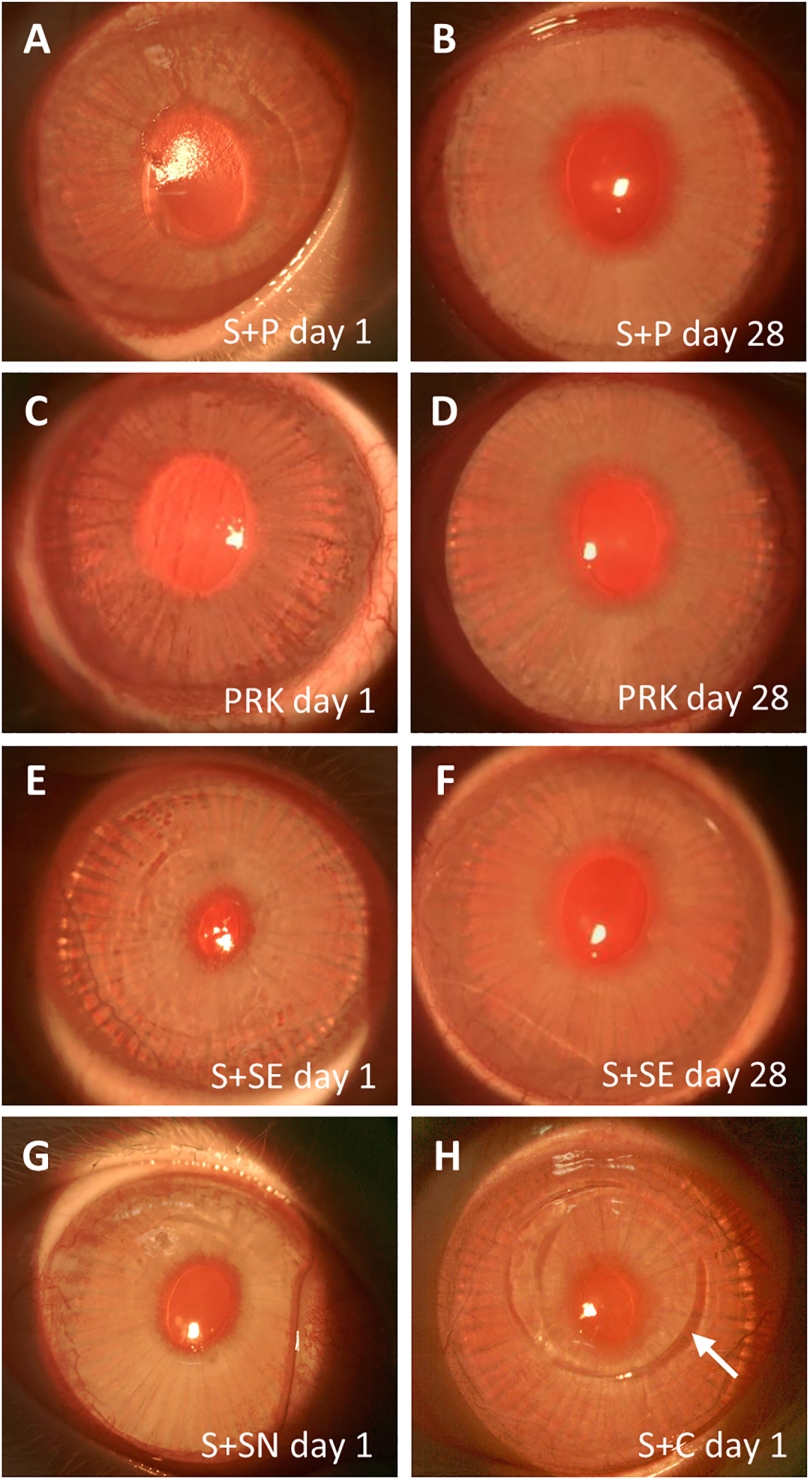
Slit lamp photographs of cornea captured after SMILE retreatment. (A) Rough surface was noted on the cornea on day 1 after PRK (S+P day 1). (B) Cornea appeared clear with smooth surface on day 28 after PRK (S+P day 28). The appearance of the corneas was comparable to those in the PRK day 1 (C) and PRK day 28 (D) groups, respectively. (E) Cornea appeared clear, but the surface became rougher on day 1 after the secondary SMILE (S+SE day 1). (F) The minor surface undulation was not seen 28 days after secondary SMILE (S+SE day 28). (G) Cornea appeared clear and the surface was smooth after secondary SMILE when the lenticule was not extracted (S+SN day 1). (H) Cornea appeared clear after flap creation by Circle pattern and subsequent stromal excimer ablation (S+C day 1). Flap side cut created by the Circle pattern, which extended beyond the small incision (arrow), was visible.

One day after SMILE retreatment, slit lamp photography showed a rough surface and slight haze on the corneas following S+P (Fig 1A). The rough surface was characteristic of the exposed anterior corneal stroma after the epithelium was scraped off and the basement membrane was subsequently ablated with an excimer laser. At day 28, the corneas appeared clear with a smooth surface (Fig 1B). Similar observation could be made on the corneas treated with PRK alone (Fig 1C and 1D). The rabbit cornea in the S+SN group showed a clear and smooth surface (Fig 1G). They also appeared clear, with minor undulations on the surface, after the secondary SMILE lenticule was extracted in S+SE day 1 group (Fig 1E). At day 28 after secondary SMILE, however, the corneal surface irregularity had resolved (Fig 1F). The corneas in S+C day 1 group were clear, although flap side cut created by the Circle pattern was still visible (Fig 1H).

### Anterior segment-optical coherence tomography

**Fig 2:**
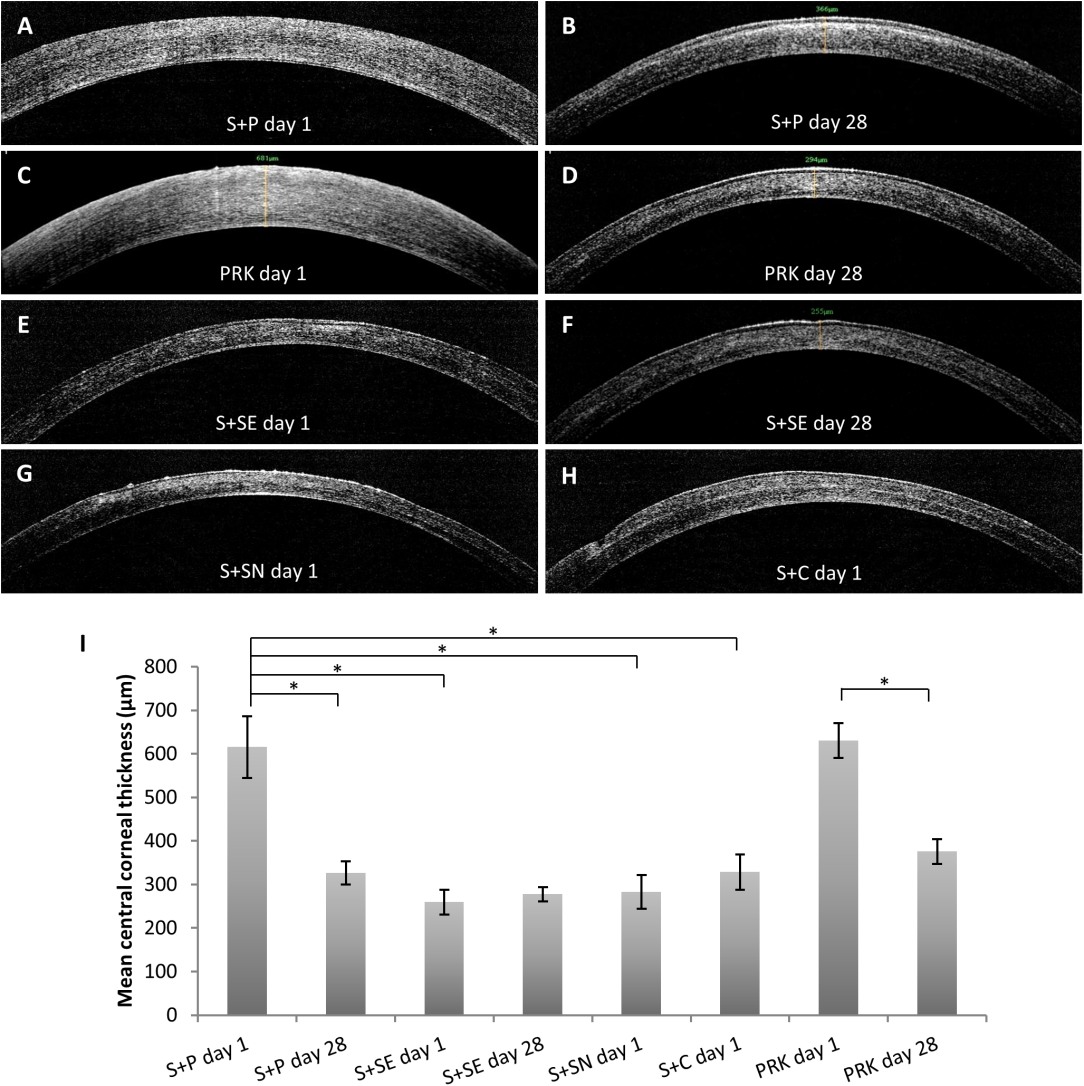
Cross-sectional visualization of cornea captured after SMILE retreatment. (A) Cornea appeared edematous on day 1 after PRK (S+P day 1). (B) Sub-epithelial stromal haze was observed on day 28 after PRK, although the cornea appeared thinner (S+P day 28). (C) Reciprocal observation in S+P day 1 could be made on cornea treated with PRK only on day 1. (D) In contrast to S+P day 28 group, stromal haze was not seen in PRK day 28 group. Corneal cap interface was hardly visible after secondary SMILE either in the groups which had the lenticule extracted (both S+SE day 1 and S+SE day 28 groups) (E and F) or the group with lenticule left not extracted (S+SN day 1 group) (G). (H) Corneal flap interface was visible as a reflective plane on day 1 after refractive enhancement utilizing VisuMax Circle software (S+C day 1 group). (I) Bar graph showing mean central corneal thickness after each retreatment method. Resulting corneal thickness was analyzed using one way ANOVA and a post hoc Tukey comparison procedure. *p<0.001.

Cross-sectional examination of the post-operative cornea demonstrated an edematous cornea, devoid of epithelial cells, at day 1 after S+P (Fig 2A). The cornea appeared re-epithelialized and significantly thinner after 28 days (from 615.7±70.9 μm to 326.7±26.8 μm; *p*<0.001), but sub-epithelial corneal haze was seen centrally (Fig 2B). We observed a similar post-operative day 1 corneal appearance in PRK day 1 group (Fig 2C), but in PRK day 28 group, the sub-epithelial corneal haze was largely absent (Fig 2D). The corneas in S+SE day 1 group (259.4±28.6 μm) appeared significantly thinner than in S+P day 1 group (*p*<0.001) (Fig 2E). Regardless of whether the lenticule was extracted or not, the corneal cap interface of the primary and secondary SMILE was hardly visible within the corneal tissue (Fig 2E–2G). In contrast, the flap interface appeared as a highly reflective layer within the cornea that underwent flap creation by the Circle software and stromal excimer ablation (Fig 2H). The resulting central corneal thickness (CCT) of each refractive enhancement method is depicted in a bar graph (Fig 2E).

### In vivo confocal microscopy

**Fig 3:**
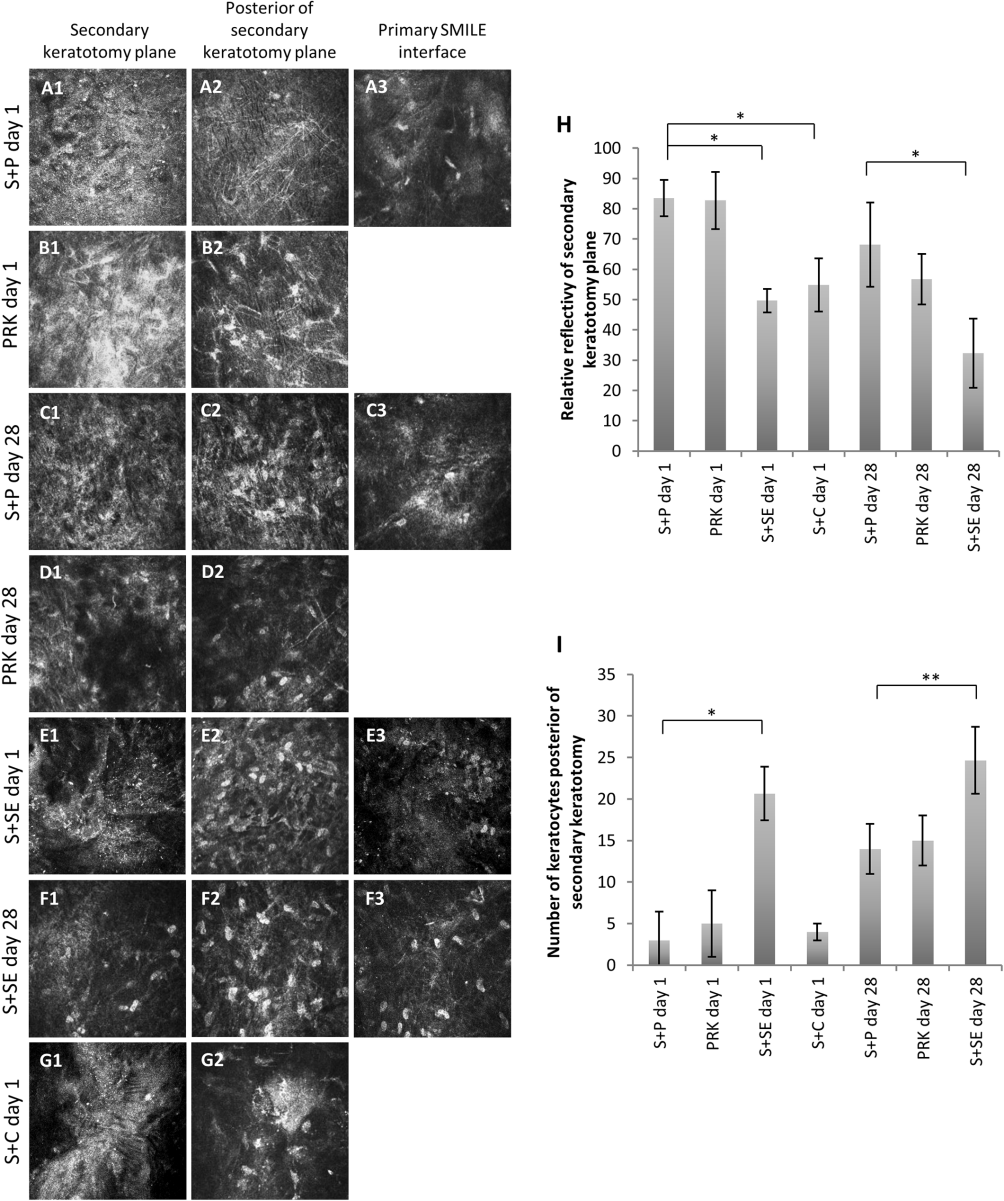
In vivo confocal micrographs of primary and secondary laser keratectomy sites and posterior plane of secondary keratectomy site captured after SMILE enhancement. Column 1 contains en-face images of secondary laser keratotomy plane after a retreatment. Column 2 shows images of in vivo confocal view 20 μm posterior of the secondary keratotomy plane. Column 3 shows images of primary SMILE interface, performed 2 weeks before the retreatment. (A) Day 1 after enhancement with PRK (S+P day 1). (B) Day 28 after enhancement with PRK (S+P day 28). (C) Day 1 after PRK only treatment (PRK day 1). (D) Day 28 after PRK only treatment. (E) Day 1 after secondary SMILE was performed anterior of the primary SMILE (S+SE day 1). (F) Day 28 after secondary SMILE was performed anterior of the primary SMILE (S+SE day 28). (G) Day 1 after excimer laser stromal ablation, following flap creation by VisuMax Circle software (S+C day 1). In this group, the flap was created from the primary SMILE cap incision; hence, the secondary keratotomy site overlapped the primary SMILE cap interface. (H) Bar graph showing mean reflectivity of the secondary laser keratotomy site after each treatment method. (I) Bar graph showing mean number of keratocytes at 20 μm posterior of the secondary keratotomy site after each treatment method. Resulting reflectivity levels and number of keratocytes were analyzed using one way ANOVA and a post hoc Tukey comparison procedure. *p<0.001 and **p<0.05.

Temporal confocal images showed a highly reflective secondary keratectomy site on day 1 after S+P (Fig 3A1). The keratectomy site was devoid of keratocytes, but was infiltrated with inflammatory cells. A few activated keratocytes were seen on the plane just posterior to the excimer laser ablation site (Fig 3A2). Several keratocytes were seen at the primary SMILE interface and the interface had become less reflective than 1 day after primary SMILE (Fig 3A3). In PRK day 1 group, the surface ablated plane was highly reflective and acellular (Fig 3B1). Activated keratocytes were present on the posterior plane to the ablation site (Fig 3B2). In S+P day 28 group, the secondary keratectomy plane and the plane posterior to it became less reflective than on post-operative day 1 (Fig 3C1 and 3C2). Some keratocytes could be observed lying in both planes (Fig 3C1 and 3C2). Quiescent keratocytes at the primary SMILE interface were visible at this time point (Fig 3C3). At day 28 after PRK only treatment, we detected a less reflective keratectomy plane than that in S+P day 28 group, although the difference in reflectivity levels was not significant (*p* = 0.364) (Fig 3D1). Some keratocytes were present on the posterior plane to the excimer laser ablated site in the PRK day 28 treatment group (Fig 3D2).

In the S+SE day 1 group, a relatively less reflective corneal cap interface of the secondary SMILE was observed (Fig 3E1). The interface was largely devoid of keratocytes, but the layer 20 μm posterior to it was marked by the presence of abundant keratocytes (Fig 3E2). Similar observation of primary SMILE interface between S+P day 1 and S+SE day 1 group could be made (Fig 3E3). At day 28 post-secondary SMILE, some keratocytes had re-populated the cap interface (Fig 3F1). Keratocytes were present in abundance in between primary and secondary cap interfaces (Fig 3F2). The primary SMILE incision plane had almost recovered at this time point, where the interfacial reflectivity was largely resolved (Fig 3F3). The flap interface of cornea in S+C day 1 group was reflective and devoid of keratocytes (Fig 3G1). Relatively low level of reflectivity was seen on the layer just posterior to the flap interface, but a few keratocytes (4±1 cells) were detected (Fig 3G2).

The reflectivity level of secondary FSL keratectomy plane after each treatment method is depicted in a bar graph (Fig 3H). Fig 3I shows the mean number of keratocytes present on the plane 20 μm posterior to the secondary FSL keratotomy plane. At day 1, it was obvious that enhancement methods utilizing excimer laser ablation resulted in fewer keratocytes than when all-FSL procedure was performed. At day 28 after surface ablation enhancement, there was a recovery in the population of keratocytes along the plane posterior to the ablation site in the S+P day 28 group (*p* = 0.01 compared to S+P day 1), but the number was still significantly less than in S+SE day 28 group (*p* = 0.01). There was no significant difference in keratocytes counted in the PRK day 1 group (5±4 cells) and the S+P day 1 group (2±3 cells) (*p* = 0.549). The trend continued on day 28, where PRK group (16±3 cells) had more keratocytes than S+P group (14±3 cells), but the difference was not significantly different.

### Immunohistochemical and TUNEL assay analysis

**Fig 4:**
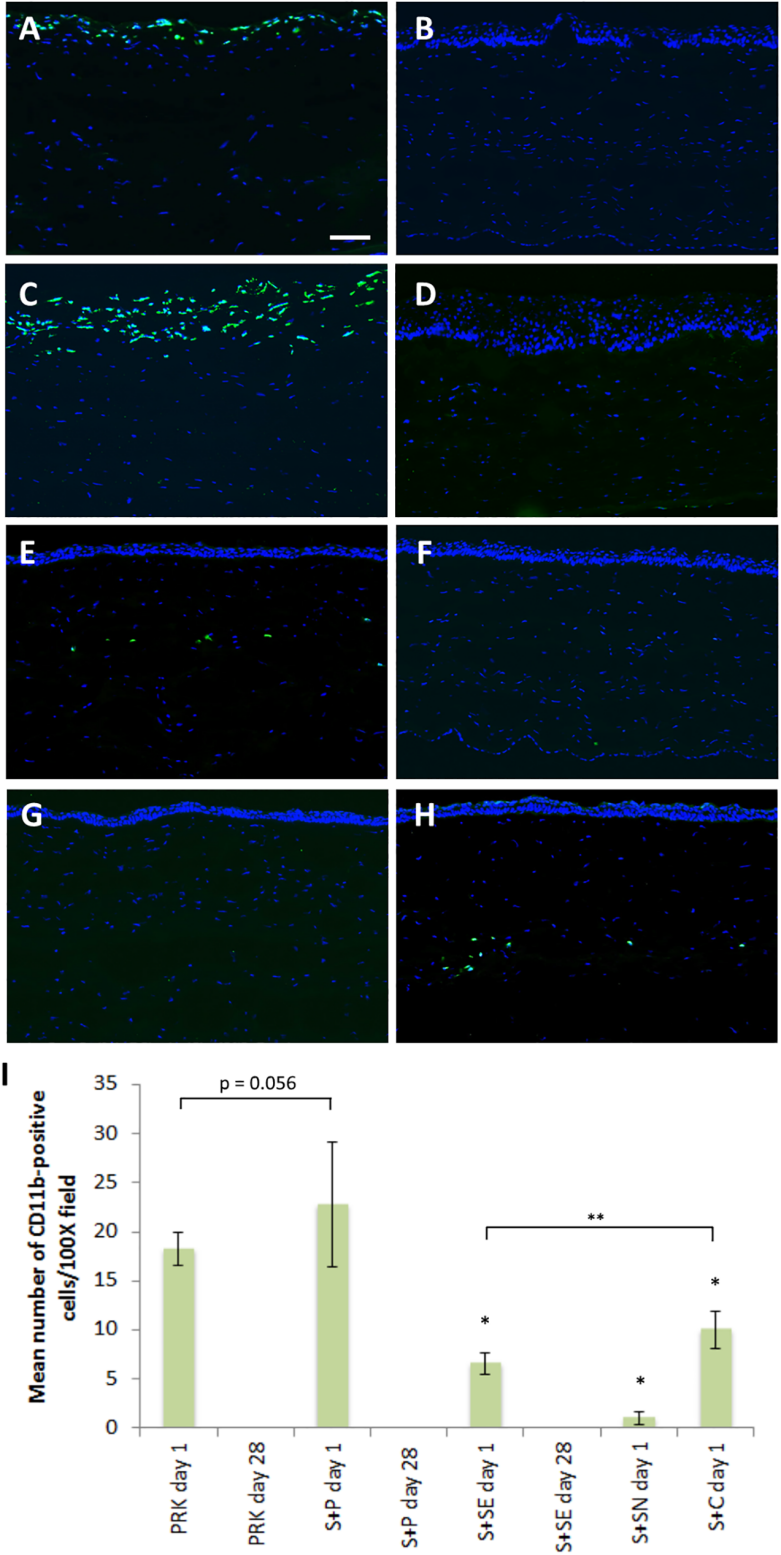
Expression of CD11b in the central cornea after SMILE retreatment. (A) Abundant of inflammatory cells was present 2–3 cells deep at the superficial layer of the exposed stroma 1 day after PRK alone (PRK day 1 group). (B) After 28 days, no inflammatory cells was seen in the stroma (PRK day 28 group). (C) CD11b-positive cells were abundantly present on day 1 in cornea treated with S+P only (S+P day 1 group). (D) Similar to PRK day 28, the inflammation was resolved on day 28 (S+P day 28 group). (E) Relatively less inflammatory cells were seen along the cap interface of the secondary SMILE after extraction of lenticule (S+SE day 1 group). (F) No CD11b staining was detected in S+SE day 28 group. (G) No inflammation occurred when the secondary SMILE lenticule was left not extracted (S+SN day 1 group). (H) Inflammatory cells were observed along the flap interface after flap creation utilizing VisuMax Circle software and subsequent stromal excimer ablation (S+C day 1 group). (I) Quantification of CD11b-positive cells in the central corneal stroma after different SMILE retreatment methods. Data were analyzed using one way ANOVA and a post hoc Tukey comparison procedure. *p<0.001 relative to S+P day 1. **p<0.05.Scale bar = 50 μm.

CD11b-positive cells, a marker for tissue inflammation [1,25], were detected along the exposed anterior stroma, about 2–3 cell layers deep at day 1 after PRK alone treatment (Fig 4A). As previously seen in our in vivo confocal images, re-epithelialization was complete at day 28 and no inflammatory cell was detectable in PRK day 28 group (Fig 4B). Similar inflammatory responses to the PRK only treatment groups was made for corneas in S+P day 1 (Fig 4C) and S+P day 28 (Fig 4D) groups. There were more CD11b-positive cells in the S+P day 1 group (23±6 cells) than in the PRK day 1 group (18±2 cells), but the difference was borderline significant (*p* = 0.056).

There were a few inflammatory cells found along the corneal cap interface of the secondary SMILE in S+SE day 1 group (Fig 4E), but none was observed at post-operative day 28 (Fig 4F) and in S+SN day 1 group (Fig 4G). CD11b-positive cells were present along the flap interface in the central cornea of rabbits in S+C day 1 group (Fig 4H). Mean number of CD11b-positive cells/100X field in the central cornea after each treatment method is depicted in a bar graph (Fig 4I). Post hoc Tukey comparison test showed that there were significantly more inflammatory cells in S+P day 1 group than S+SN day 1, S+SE day 1 or S+C day 1 groups (all *p*<0.001).

**Fig 5:**
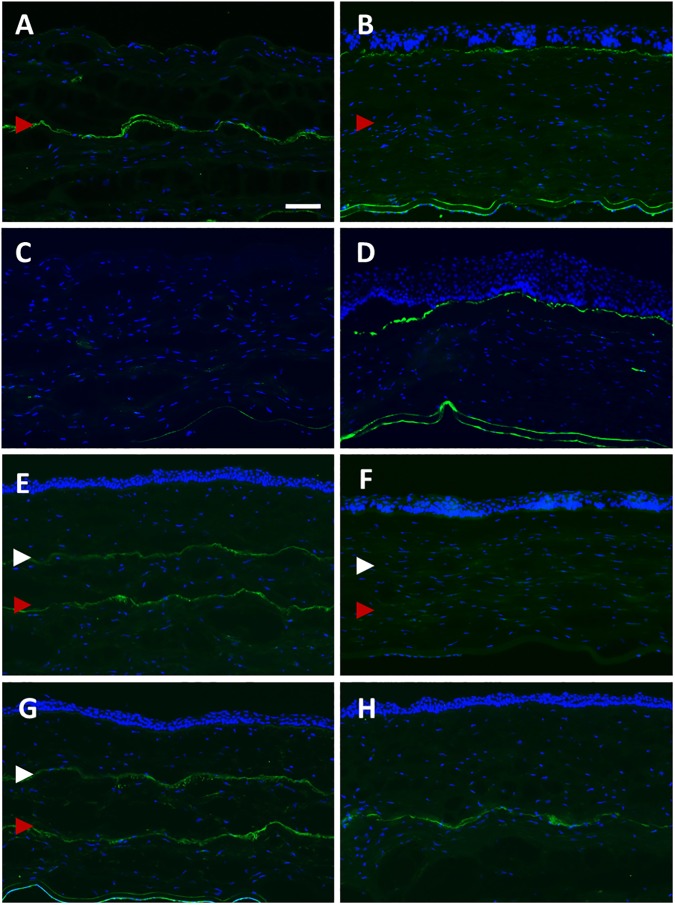
Expression of fibronectin in the central cornea after SMILE retreatment. (A) Fibronectin was not present at the superficial layer of the exposed stroma on day 1 after enhancement with PRK (S+P day 1 group), but appeared relatively strong along the primary SMILE interface located in the posterior stroma (red arrowhead). (B) On day 28, fibronectin was localized in the sub-epithelial layer of the cornea (S+P day 28 group). (C) Fibronectin staining was not present in any layer of the stroma on day 1 after PRK only treatment (PRK day 1 group). (D) Similar to S+P day 28, the staining was localized in the sub-epithelium in PRK day 28 group. (E) The interface resulted from the primary SMILE (red arrowhead) and from the secondary SMILE (white arrowhead) expressed fibronectin (S+SE day 1 group). (F) Post-secondary SMILE day 28, no expression of fibronectin was observed in the stroma (S+SE day 28 group). (G) In S+SN day 1 group, both primary SMILE interface and secondary SMILE cap cut expressed fibronectin. (H) Staining was seen along the flap interface in S+C day 1 group. Scale bar = 50 μm.

Fibronectin was not expressed in the superficial stromal layer of cornea in the S+P day 1 group, but appeared relatively strong along the laser dissection plane located in the posterior stroma, which was produced by the primary SMILE. (Fig 5A). At day 28, the primary SMILE cap interface no longer expressed fibronectin, but the staining was seen along the sub-epithelium (Fig 5B). No fibronectin was detected in PRK only day 1 group (Fig 5C), but similar to S+P day 28, fibronectin was localized in the sub-epithelium and expressed in similar intensity at day 28 (Fig 5D). In S+SE day 1 group, fibronectin was present in the posterior cap interface, resulted from the primary SMILE, and also in the anterior cap interface, created by the secondary SMILE (Fig 5E). Similar observation could be made for S+SN day 1 group, where the antibody labelling was along both the anterior and the posterior SMILE interfaces (Fig 5G). Because the enhancement correction was only -1.00D, which was equivalent to the removal of about 13-μm thick tissue, the anterior and posterior cuts of the refractive lenticule of secondary SMILE, which express fibronectin, could hardly be discerned by immunostaining. Fibronectin was no longer expressed in either interface at day 28 after secondary SMILE (Fig 5F). Fibronectin was localized along the flap interface in the central cornea of rabbits in S+C day 1 group (Fig 5H).

**Fig 6:**
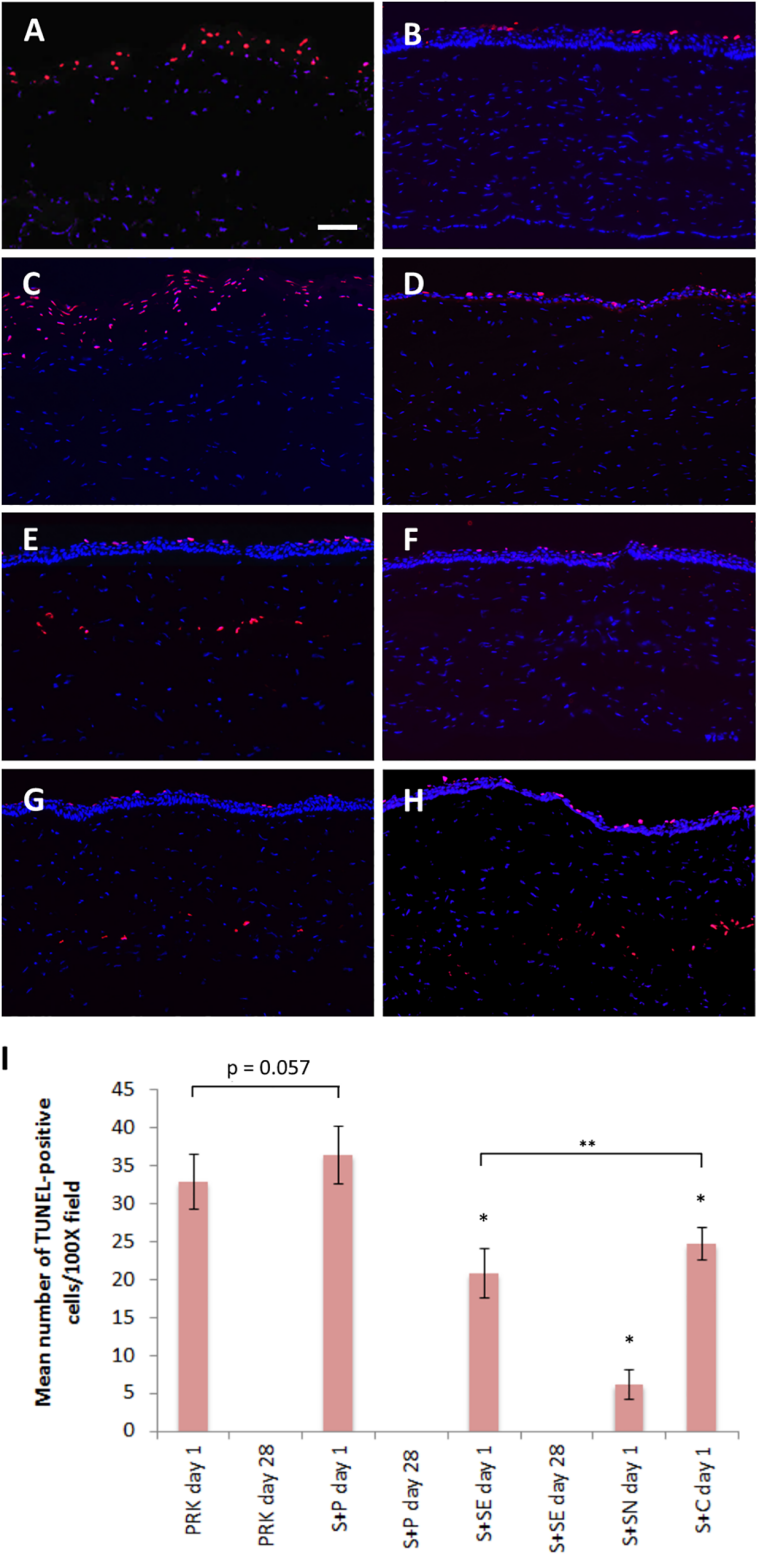
Apoptotic cells in the central cornea after SMILE retreatment. (A) Abundant of TUNEL-positive cells was present at the superficial layers of the exposed stroma on day 1 after PRK alone treatment (PRK day 1 group). (B) TUNEL staining was negative in the stroma after 28 days (PRK day 28 group). (C) Similar staining pattern to PRK day 1 group could be seen in S+P day 1 group. (D) On day 28 post-enhancement with surface ablation, no apoptotic cells was visible in the stroma (S+P day 28 group). (E) Relatively less apoptotic cells were seen along the cap interface of the secondary SMILE on post-enhancement day 1 (S+SE day 1 group). (F) No TUNEL-positive cells was observed along the cap interface after 28 days (S+SE day 28 group). (G) Some apoptotic cells were present although the lenticule was left not extracted in S+SN day 1 group. (H) Apoptotic cells were observed on day 1 along the flap interface after flap creation using VisuMax Circle software and subsequent stromal excimer ablation (S+C day 1 group). (E) Quantification of TUNEL-positive cells in the central corneal stroma after different SMILE retreatment methods. Data were analyzed using one way ANOVA and a post hoc Tukey comparison procedure. *p<0.001 relative to S+P day 1 group. **p<0.001. Scale bar = 50 μm.

Ki-67 staining was negative in the corneal stroma regardless of the type of enhancement method performed at any time point (S2A–S2H Fig). Apoptotic cells were detected along the exposed anterior stroma, 2–3 cell layers deep at day 1 after PRK alone treatment (Fig 6A). No TUNEL-positive cells were found in the corneal stroma after the stroma was re-epithelialized at day 28 after PRK alone (Fig 6B). The staining pattern was similar to the S+P day 1 and day 28 groups (Fig 6C and 6D). There were more TUNEL-positive cells in the S+P day 1 group (36±4 cells) than in the PRK day 1 group (32±4 cells), but the difference was borderline significant (p = 0.057).

TUNEL staining could be observed along the corneal cap interface of the secondary SMILE in S+SE day 1 group (Fig 6E) and a few apoptotic cells were also observed even when the lenticule was not extracted (Fig 6G). In S+SE day 28 group, TUNEL staining was negative in the corneal stroma (Fig 6F). Apoptotic cells could be seen along the flap interface in the central cornea of rabbits in S+C day 1 group and in some regions were 2–3 cell layers deep (Fig 6H). Mean number of TUNEL-positive cells in the central corneal stroma after each treatment method is depicted in a bar graph (Fig 6I). Post hoc Tukey comparison test revealed a significant difference in the number of apoptotic cells in S+P day 1 group than S+SN day 1, S+SE day 1 or S+C day 1 groups (all *p*<0.001). Significant difference was also detected between S+SE day 1 and S+SN day 1 groups (*p*<0.001).

## Discussion

In this study, we assessed 3 different methods that could possibly be applied for SMILE enhancement, namely surface ablation, performing a secondary SMILE just anterior to primary SMILE, and creating a corneal flap using VisuMax Circle pattern followed by stromal excimer ablation. Each SMILE retreatment method resulted in unique tissue responses. Performing secondary SMILE offered advantages, such as minimal inflammation and cell death, as well as maintaining a ‘flap-less’ surgery, over other procedures. However, depending on the degree of enhancement, the lenticule may become too thin to be extracted and the procedure may be more difficult to perform than converting SMILE cap into a flap and surface ablation. Stromal excimer ablation following SMILE cap to flap conversion resulted in tissue responses similar to those following a femto-LASIK. Surface ablation was the most simplest enhancement technique to perform, but the surgery resulted in excessive inflammation and corneal haze, necessitating the use of MMC.

The removal of corneal epithelium by scrapping and subsequent surface ablation by excimer laser is known to trigger an excessive presence of CD11b- and TUNEL-positive cells [23]. The excimer laser results in the release of pro-inflammatory cytokines, such as IL-1 and TNF-α, that bind to receptors on the surviving stromal keratocytes, stimulating the release of chemokines to recruit inflammatory cells at the site of injury [26,27]. The release of pro-inflammatory cytokines can also be followed by apoptosis in the exposed keratocytes [28]. In our study, since the attempted refractive correction was relatively small (-1.00D), we observed more number of inflammatory and apoptotic cells induced by SMILE enhancement by PRK compared to PRK alone treatment. In addition, on AS-OCT, there was relatively more intense sub-epithelial haze developed in the S+P group compared to the PRK alone group at day 28. The difference is possibly due to the secondary surgical procedure in the S+P group. Repeated corneal injury induced by PRK without post-operative anti-scarring agents has been shown to cause chronic inflammation and aberrant wound healing [29].

Fibronectin was not expressed in the S+P day 1 and PRK day 1 groups, but was expressed in similar intensity in both S+P day 28 and PRK day 28 groups, and its expression was concentrated in the sub-epithelial layer, consistent with a previously reported rabbit model of PRK [30]. Activity of fibronectin is normally upregulated during the corneal epithelial cell migration process by providing an adherent surface in the absence of basement membrane or anchoring fibrils after injury, including after PRK [31].

The Circle pattern D has been reported to successfully convert pockets into flaps [19,20]. Here, we used the same Circle pattern and subsequently performed a stromal excimer ablation as one of the alternatives of SMILE enhancement. As expected, after a low myopic correction, the tissue response to excimer laser ablation and ReLEx was similar [1]. There was no difference in the intensity of the reflective keratectomy plane, expression of fibronectin and CCT between CE and SSE groups. However, we found significantly less inflammatory cells and apoptotic cells between S+C and S+SE groups, albeit the difference was numerically small; 6 ± 1 inflammatory cells in SSE compared to 10 ± 2 cells in S+C (*p* = 0.001) and 21 ± 3 apoptotic cells in S+SE compared to 25 ± 2 cells in S+C (*p*<0.001). This difference could be attributed to the creation of flap, a surgical step that induces epithelial disruption and corneal inflammatory response [1]. With a reduced incision size, we found that SMILE procedure induced less inflammation.

The present results suggest that performing a secondary SMILE procedure may be more advantageous over the other enhancement methods in terms of generating a more structurally stable cornea, minimal wound healing response, and preservation of keratocytes. This is in contrast with excimer laser ablated stroma. The presence of these keratocytes along the FSL incision plane is important in regulating the post-surgical wound healing [32]. Keratocytes have also been shown to be responsible in modulating extracellular matrix synthesis and cytoskeleton organization [33].

However, depending on the degree of enhancement correction, it may be technically easier to perform S+P or S+C than S+SE when retreatment is indicated. One diopter of correction is equivalent to about 13 μm of central thickness of the lenticule [34]. Therefore, it would pose a significant challenge to extract a lenticule with thickness less than 13 μm, without an increased risk of tearing the lenticule. Also depending on the depth of the primary SMILE cap, there may not be sufficient anterior stroma to create another lenticule due to software limitation. Another issue of performing S+SE enhancement is the possible interference between the new interface and the existing interface. The minimum and maximum depths of the SMILE cap at the time of the study were limited to 110 μm and 160 μm, respectively. If the primary SMILE cap interface is created at the depth of 110–130 μm, the limitation of the incision depth renders performing a secondary SMILE anterior to the primary procedure impossible. In this scenario, another option would be to perform a sub-cap-lenticule-extraction, in which the interface of the primary SMILE procedure becomes the superior plane of the new lenticule and the FSL cuts only the inferior plane and the side cut of the new lenticule. This would create another lenticule posterior to the previous one [35].

In our study, we set the initial SMILE cap interface at a depth of 160 μm to allow enough anterior stroma for performing another SMILE with 110 μm-deep cap interface. Depending on the degree of enhancement correction, ±2SD of the accuracy of the VisuMax femtosecond laser system (SD range from 9.5 to 10.9 μm) [36], and the depth of primary SMILE cap into consideration, a new SMILE could be performed if the patient requires a correction of at least -1.00D and the previous SMILE was created at the depth of at least 140 μm. If the primary cap was placed at the depth of 160 μm, the maximum myopic enhancement that could be carried out is -2.25D, putting the secondary cap at the depth of 110 μm, with a safety margin of 20 μm between the interfaces.

Ivarsen and colleagues reported 5 cases with topography-guided PRK after SMILE [21]. Among the 5 cases, three eyes that did not receive peri-operative MMC treatment developed trace to grade 2 corneal haze. Our animal study showed that SMILE enhancement by PRK, although the attempted refractive correction was only -1.00D, induced more inflammation, apoptosis and sub-epithelial haze development than when the corneas were treated with PRK alone. This suggests that MMC application has to supplement SMILE enhancement by surface ablation in order to modulate better wound healing response.

Initial results of hyperopic ReLEx correction have been reported, but have shown variable efficacy [37]. If a hyperopic enhancement is indicated, S+C may be the most viable option to be performed, followed by surface ablation with MMC depending on the residual stromal bed. The formation of lamellar ring in Circle option allows the creation of a lamellar plane that is larger than the original cap cut, which is ideal for a hyperopic correction.

In conclusion, all 3 methods proposed in the current study are viable options when an enhancement is required after primary SMILE procedure. However, the decision to employ one method over the other may depend on several factors (Table 1), such as depth of primary SMILE cap, post-primary procedure residual stromal bed thickness, refractive correction required, whether a hyperopic or myopic enhancement is required, desired acceptable post-operative recovery time, and if the patient wants to maintain a ‘flap-less’ procedure for vocational reasons. Further studies in human patients are needed to confirm our current findings.

**Table 1:** Factors that may determine the selection of a SMILE enhancement technique over the other.

	SA+MMC	S+SE	S+C	SCLE
Enhancement correction required	Any	≥ -1.00D	Any	Any
Depth of primary SMILE cap	Any	≥140 μm	≤120 μm	≤130 μm
Post-primary SMILE residual stromal bed thickness	Any	>250 μm	≥300 μm	>250 μm
Hyperopic or myopic enhancement	Both	Myopic only	Both	Myopic only
Flap or maintain flap-less	Flap-less	Flap-less	Flap	Flap-less
Wound recovery time	Slow	Fast	Intermediate	Fast

SA = surface ablation

MMC = mitomycin C

S+SE = new SMILE anterior to previous SMILE procedure

S+C = flap creation by VisuMax Circle, followed by excimer stromal ablation

SCLE = sub-cap lenticule extraction (described by Donate et al. [33])

## Supporting information

S1 Fig Summary of the animal experimental design. S+P = SMILE enhancement by surface ablation. S+SE = secondary SMILE was performed anterior of the primary SMILE. S+SN = secondary SMILE was performed anterior of the primary SMILE, but the lenticule was not extracted. S+C = excimer laser stromal ablation, following flap creation by VisuMax Circle software. PRK = photorefractive keratectomy.
(TIF)

S2 Fig Expression of Ki-67 in the central cornea after SMILE retreatment. Ki-67 was not expressed in the central corneal stroma after any enhancement method. (A) Day 1 after PRK only treatment (PRK day 1). (B) Day 28 after PRK only treatment (PRK day 28). (C) Day 1 after SMILE enhancement by surface ablation (S+P day 1). (D) Day 28 after enhancement by surface ablation (S+P day 28). (E) Day 1 after secondary SMILE was performed anterior of the primary SMILE (S+SE day 1). (F) Day 28 after secondary SMILE was performed anterior of the primary SMILE (S+SE day 28). (G) Secondary SMILE was performed anterior of the primary SMILE, but the lenticule was not extracted (S+SN day 1). (H) Day 1 after excimer laser stromal ablation, following flap creation by VisuMax Circle software (S+C day 1). Scale bar = 50 μm.
(TIF)
