# Supplementary figures and images for: Retreatment strategies following Small Incision Lenticule Extraction (SMILE): In vivo tissue responses
Source: PLoS One. 2017 Jul 14;12(7):e0180941. doi: 10.1371/journal.pone.0180941 (PMC5510831; doi:10.1371/journal.pone.0180941)

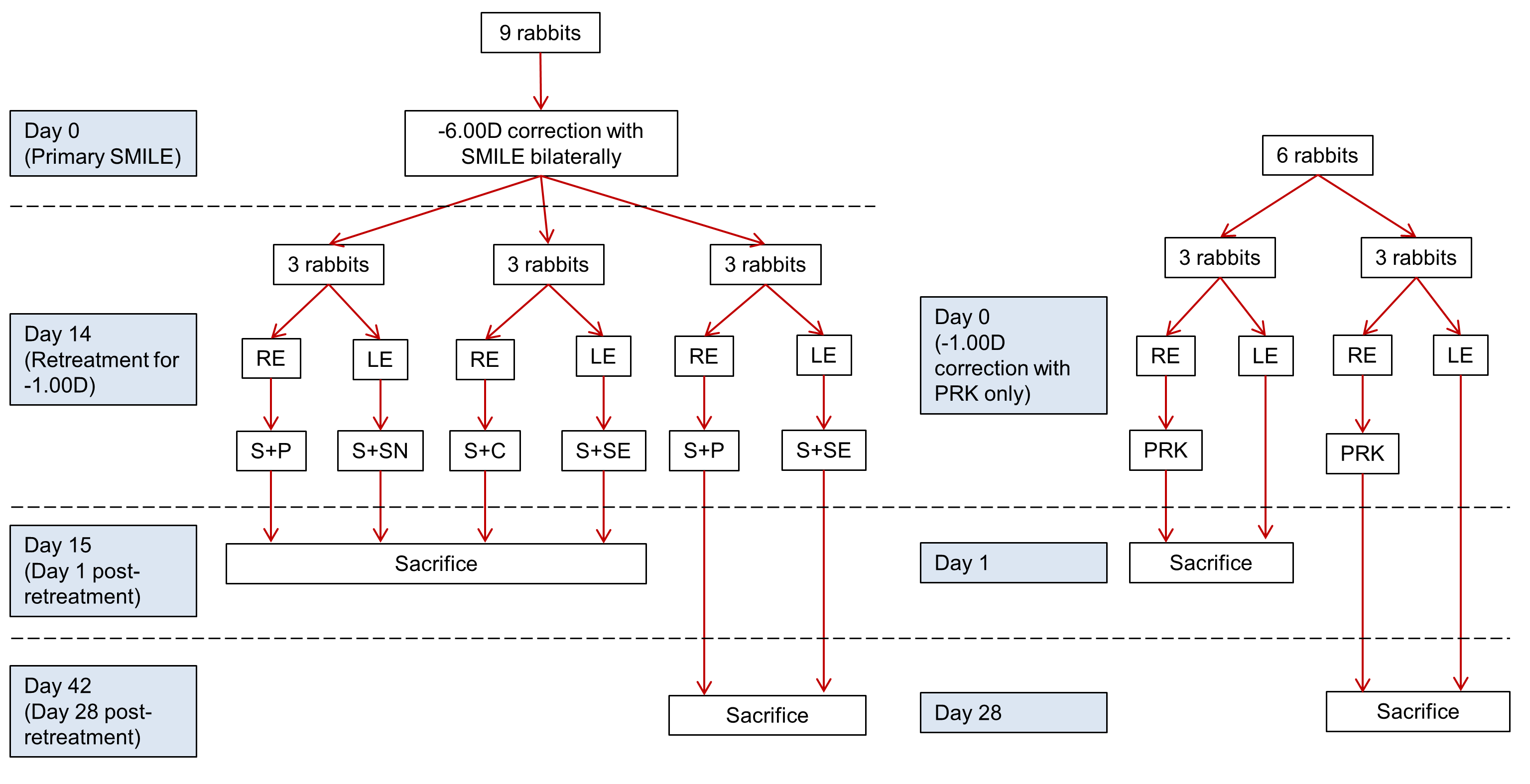

Supplement: S1 Fig — S+P = SMILE enhancement by surface ablation. S+SE = secondary SMILE was performed anterior of the primary SMILE. S+SN = secondary SMILE was performed anterior of the primary SMILE, but the lenticule was not extracted. S+C = excimer laser stromal ablation, following flap creation by VisuMax Circle software. PRK = photorefractive keratectomy. (TIF) [file pone.0180941.s001.tif]

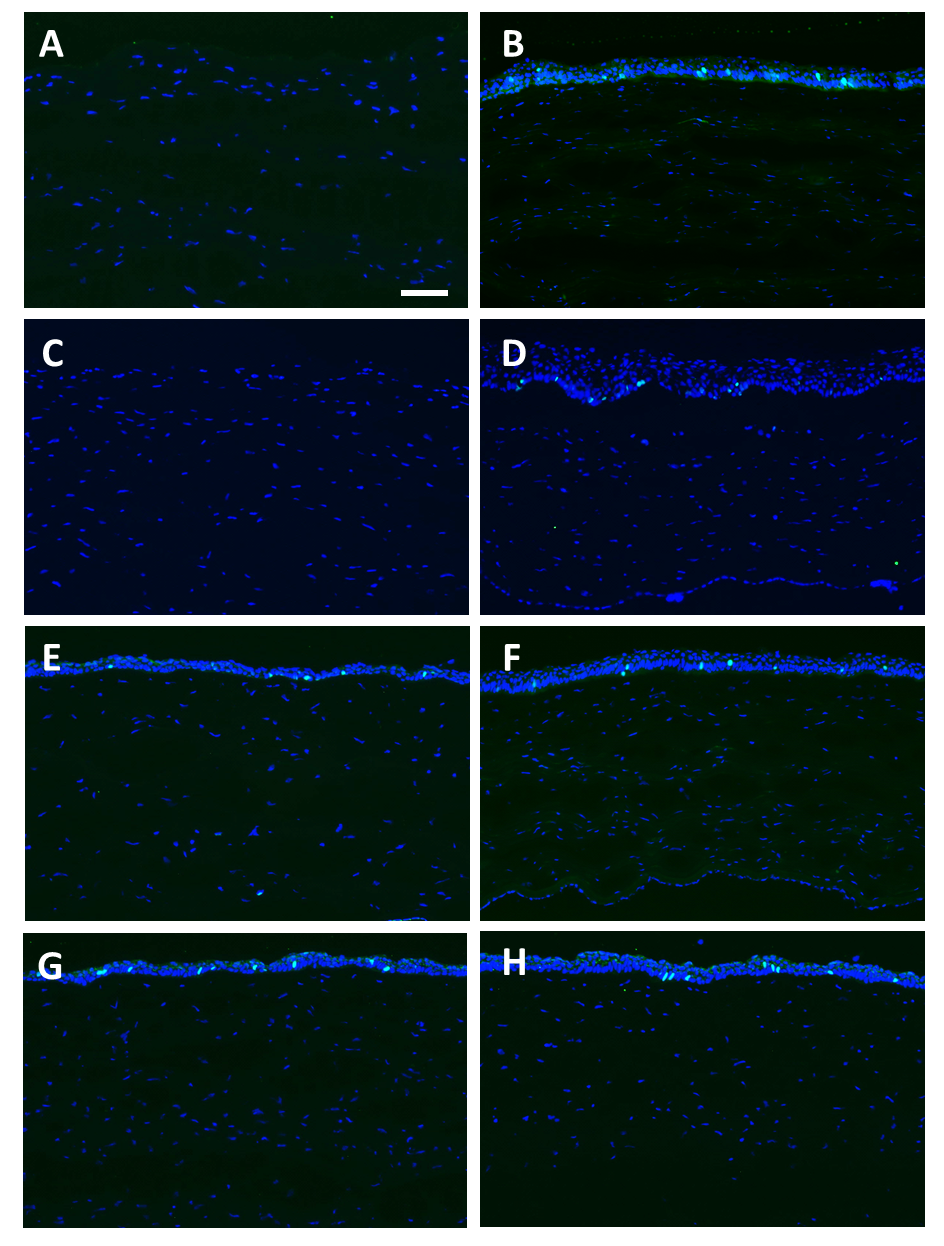

Supplement: S2 Fig — Ki-67 was not expressed in the central corneal stroma after any enhancement method. (A) Day 1 after PRK only treatment (PRK day 1). (B) Day 28 after PRK only treatment (PRK day 28). (C) Day 1 after SMILE enhancement by surface ablation (S+P day 1). (D) Day 28 after enhancement by surface ablation (S+P day 28). (E) Day 1 after secondary SMILE was performed anterior of the primary SMILE (S+SE day 1). (F) Day 28 after secondary SMILE was performed anterior of the primary SMILE (S+SE day 28). (G) Secondary SMILE was performed anterior of the primary SMILE, but the lenticule was not extracted (S+SN day 1). (H) Day 1 after excimer laser stromal ablation, following flap creation by VisuMax Circle software (S+C day 1). Scale bar = 50 μm. (TIF) [file pone.0180941.s002.tif]
